# Systemic Therapy for Cervical Cancer with Potentially Regulatable Oncolytic Adenoviruses

**DOI:** 10.1371/journal.pone.0002917

**Published:** 2008-08-13

**Authors:** Anna Kanerva, Sergio Lavilla-Alonso, Mari Raki, Lotta Kangasniemi, Gerd J. Bauerschmitz, Koichi Takayama, Ari Ristimäki, Renee A. Desmond, Akseli Hemminki

**Affiliations:** 1 Cancer Gene Therapy Group, Molecular Cancer Biology Program and Transplantation Laboratory and Finnish Institute for Molecular Medicine, University of Helsinki, Helsinki, Finland; 2 Department of Obstetrics and Gynecology, Helsinki University Central Hospital (HUCH), Helsinki, Finland; 3 Transplantation Laboratory, HUSLAB, Helsinki University Central Hospital (HUCH), Helsinki, Finland; 4 Department of Obstetrics and Gynecology, Heinrich-Heine University, Duesseldorf, Germany; 5 Research Institute for Diseases of the Chest, Kyushu University, Fukuoka, Japan; 6 Department of Pathology, HUSLAB, Helsinki University Central Hospital (HUCH), and Genome-Scale Biology Program, University of Helsinki, Helsinki, Finland; 7 Comprehensive Cancer Center, Biostatistics and Bioinformatics Unit, University of Alabama at Birmingham, Birmingham, Alabama, United States of America; University of Florida, United States of America

## Abstract

Clinical trials have confirmed the safety of selectively oncolytic adenoviruses for treatment of advanced cancers. However, increasingly effective viruses could result in more toxicity and therefore it would be useful if replication could be abrogated if necessary. We analyzed viruses containing the cyclooxygenase-2 (Cox-2) or vascular endothelial growth factor (VEGF) promoter for controlling replication. Anti-inflammatory agents can lower Cox-2 protein levels and therefore we hypothesized that also the promoter might be affected. As Cox-2 modulates expression of VEGF, also the VEGF promoter might be controllable. First, we evaluated the effect of anti-inflammatory agents on promoter activity or adenovirus infectivity *in vitro*. Further, we analyzed the oncolytic potency of the viruses *in vitro* and *in vivo* with and without the reagents. Moreover, the effect of on virus replication was analyzed. We found that RGD-4C or Ad5/3 modified fibers improved the oncolytic potency of the viruses *in vitro* and *in vivo*. We found that both promoters could be downregulated with dexamethasone, sodium salicylate, or salicylic acid. Oncolytic efficacy correlated with the promoter activity and *in vitro* virus production could be abrogated with the substances. *In vivo*, we saw good therapeutic efficacy of the viruses in a model of intravenous therapy of metastatic cervical cancer, but the inhibitory effect of dexamethasone was not strong enough to provide significant differences in a complex *in vivo* environment. Our results suggest that anti-inflammatory drugs may affect the replication of adenovirus, which might be relevant in case of replication associated side effects.

## Introduction

The pathogenesis of cervical cancer is characterized by persistent infection with a high-risk human papillomavirus (HPV), generally accepted as required for cervical cancer initiation. In a fraction of patients, HPV infection progresses from dysplasia and carcinoma *in situ* to invasive cancer and metastatic disease [1]. Only a few viral strains are specifically responsible for cervical neoplasms, of which HPV16 accounts for more than one-half of reported cases. Unfortunately, neither improvements in surgery nor radiotherapy have significantly decreased mortality of patients with advanced, recurrent, or metastatic disease. The American Cancer Society estimates about 9 700 new cases and 3 700 deaths in cervical cancer in 2006 [2]. However, cervical cancer remains the leading cause of gynecological cancer mortality worldwide with over 270 000 deaths in 2002 [3].

Adenoviral gene therapy has been proposed as a novel treatment alternative for advanced cancer [4]. However, effective tumor transduction continues to be the limiting step for achieving clinical results. Oncolytic adenoviruses might prove useful in this regard [5]. These viruses have a cytolytic nature, *i.e.* the replicative life cycle of the virus results in host cell destruction. Modifications in the viral genome reduce replication in normal tissues, while tumor cells continue to allow productive replication leading to cancer cell lysis (oncolysis). Type I oncolytic adenoviruses feature loss-of-function mutations in the virus genome, which are compensated by mutations in cancer but not normal cells. This can be achieved by incorporating deletions in the early adenoviral genes resulting in mutant E1 proteins unable to bind cellular proteins necessary for viral replication in normal cells, but not in cancer cells. In type II viruses, tumor or tissue specific promoters replace endogenous viral promoters such as the E1A promoter, to restrict viral replication to target tissues expressing the promoter.

Although clinical trials have confirmed the safety of oncolytic adenoviruses for treatment of advanced cancers [6]–[9], most trials have featured relatively attenuated viruses. Thus, increasingly effective agents could result in more toxicity and therefore it would be useful if replication could be abrogated if necessary. Gene expression from certain promoters can be regulated. For example, the early growth response gene 1 (*egr*-1) enhancer/promoter, has been used as a regulatable promoter for specific expression of HSV-TK in glioma cells and can be induced by radiation [10]. Another regulation strategy is the use of hypoxia-inducible promoters [11]. Further, regulation can be achieved with chemically inducible promoters. For example, a tetracycline-activated promoter can be used to regulate gene expression and subsequent protein production by oral tetracycline. Withdrawal of the drug rapidly abrogates gene expression [12].

Cox-2 is the rate-limiting enzyme in prostaglandin synthesis, and it is involved in the control of inflammatory reactions in response to injury or infection. Use of non-steroidal anti-inflammatory drugs (NSAIDs) has indicated that the activity and also level of the Cox-2 protein can be regulated. Although other factors besides promoter activity often have a role in protein expression levels, studies have shown that activity of the Cox-2 promoter correlates well with protein expression [13]. Further, the activity of the Cox-2 promoter in most healthy normal tissues is low, unless it is induced by growth factors (such as VEGF), cytokines or tumor specific factors [14]. With regard to adenoviruses, the most relevant organ for toxicity is the liver. Therefore, it is useful that hepatic expression of Cox-2 is low [15], [16]. Cox-2 may have a role in the carcinogenesis of many epithelial cancers, and expression levels have been linked to tumor invasiveness and angiogenesis [14]. These reasons have led to utilization of the Cox-2 promoter as a tumor specific promoter for cancer specific expression [15]–[20].

VEGF has an important role in the induction of tumor-associated angiogenesis, as it is a mediator of endothelial cell proliferation, differentiation, and vascular permeability [21]. VEGF is widely expressed during tumorigenesis and it is detected in most malignant epithelial tumors [18], [19], [21] Cox-2 or growth factors like TGF-β1 can regulate VEGF [22], [23]. Further, bulky solid tumor masses contain hypoxic areas, which feature high levels of the HIF-1 transcription factor, which in turn induces VEGF and Cox-2 expression [23]. Thus, the regulation and expression of Cox-2 and VEGF are linked. Consequently, it is not completely surprising that also the VEGF promoter has shown utility for tumor specific expression [18], [19], [24]. A heretofore unexplored possibility is regulation of the Cox-2 and VEGF promoters by anti-inflammatory agents. This might offer a safety switch in case of promoter mediated side effects in clinical trials.

Clinical cervical cancer samples express high levels of Cox-2, while it is undetectable in the normal epithelial lining of the cervix. Further, there is a progressive increase in Cox-2 levels depending on disease stage, and also tumor size. Cox-2 expression is also a negative predictive factor for survival [25], [26]. With regard to VEGF and cervical cancer, a high pretreatment level has been found to associate with large tumors, stromal invasion and pelvic lymph node metastasis. VEGF expression also correlates with poor prognosis [27]–[29]. Therefore, both promoters are appealing candidates for cervical cancer specific gene therapy approaches.

Sodium salicylate and salicylic acid are NSAIDs, which enzymatically inhibit Cox-2. Further, it has been reported that these substances decrease Cox-2 mRNA levels, which could be mediated through modulation of promoter activity [13], [30]. Dexamethasone is an anti-inflammatory steroid which inhibits expression of both Cox-2 mRNA and protein [30]. Further, post-transcriptional mRNA destabilization may be an important mechanism in the action of dexamethasone [31]. TGF-β1 is a peptide growth factor and anti-inflammatory cytokine, which is produced by many cells, but is found most concentrated in mammalian platelets. It can modulate by for instance cell proliferation and differentiation, angiogenesis and metastasis, and has been shown to have an effect on Cox-2 levels [32].

We hypothesized that it may be possible to reduce adenovirus replication with pharmacological intervention. Specifically, we analyzed oncolytic viruses containing the cyclooxygenase-2 (Cox-2) or the vascular endothelial growth factor (VEGF) promoter controlling expression of *E1A*, and evaluated the effect of anti-inflammatory reagents [sodium salicylate, dexamethasone, salicylic acid and transforming growth factor-β1 (TGF-β1)] on oncolysis and replication *in vitro* and *in vivo* efficacy. As controls, we included a Retinoblastoma (Rb)-p16 pathway selective Δ24-based type I oncolytic virus [33] and a wild type adenovirus. Further, as it has become evident that a major determinant of the efficacy of replicating adenoviruses is gene delivery efficacy [34], we utilized fiber modified, infectivity enhanced viruses.

## Results

### Infectivity of human cervical cancer cell lines *in vitro*

**Figure 1:**
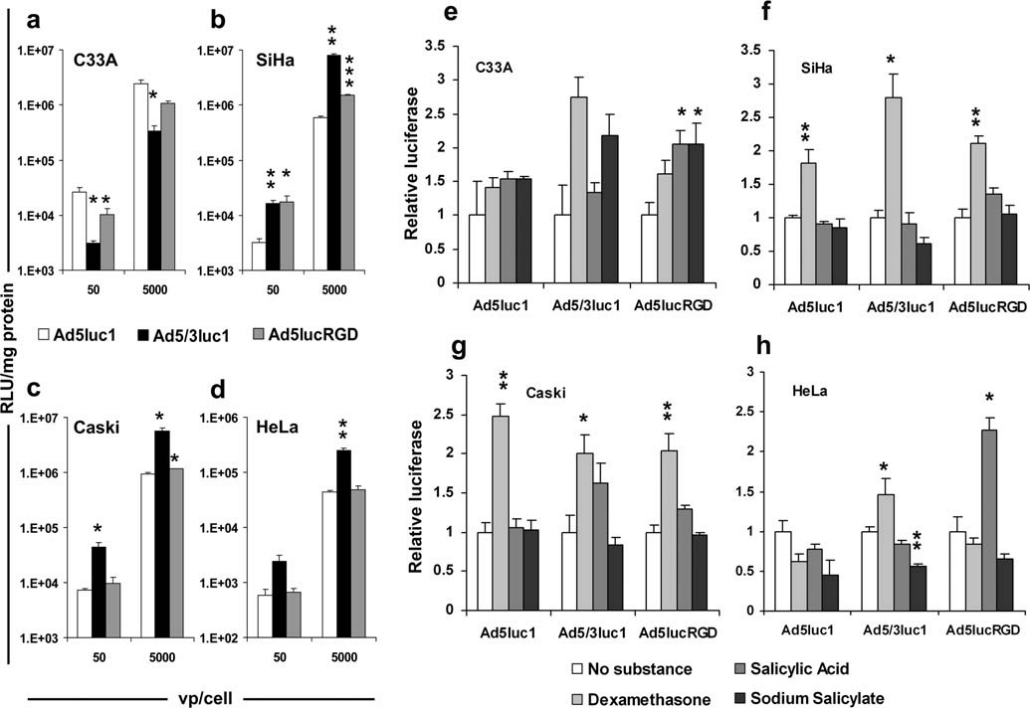
Infectivity of cervical cancer cells by adenoviral vectors with fiber knob modifications and the effect of anti-inflammatory reagents on transduction efficacy. (a–d) Cell lines were infected with Ad5luc1, Ad5/3luc1, and Ad5lucRGD. Luciferase activity is expressed as relative light units (RLU) normalized for total protein concentration. (e–h) The effect of anti-inflammatory reagents on transduction efficacy of capsid modified adenoviruses. Cells were infected in the presence of substances. The value without reagents was set at 1, and relative luciferase values are shown. Each point represents the mean of three experiments±standard error. *P<0.05, **P<0.01, ***P<0.0001 *versus* Ad5luc1.

Cervical cancer cell lines C33A, SiHa, Caski and HeLa were infected with isogenic luciferase expressing viruses featuring either the adenovirus serotype 5 capsid (Ad5luc1), a chimeric capsid with the knob domain from serotype 3 (Ad5/3luc1) or the RGD-4C capsid modification (Ad5lucRGD). In three out of four cell lines, infection with Ad5/3luc1 resulted in 6 to 14-fold higher luciferase expression in comparison to Ad5luc1 (5 000 viral particle (vp)/cell, Fig. 1b–d). However, with C33A cells, which feature high expression of the coxsackie-adenovirus receptor [19], [35], Ad5luc1 was most effective (7-fold, Fig. 1a). Ad5lucRGD did not increase the infectivity of cervical cancer cells *in vitro*, except in SiHa cells (2.5 to 5.5-fold enhancement, Fig. 1b).

### The effect of anti-inflammatory reagents on transduction efficacy of capsid modified adenoviruses

Cervical cancer cell lines were infected with capsid modified adenoviruses in the presence of substances. As shown in Fig. 1e–h, dexamethasone increased the transduction efficacy with all the viruses on SiHa and Caski cell lines. Other analyzed substances had only minor effect.

### Regulation of Cox-2 and VEGF promoters with anti-inflammatory reagents

**Figure 2:**
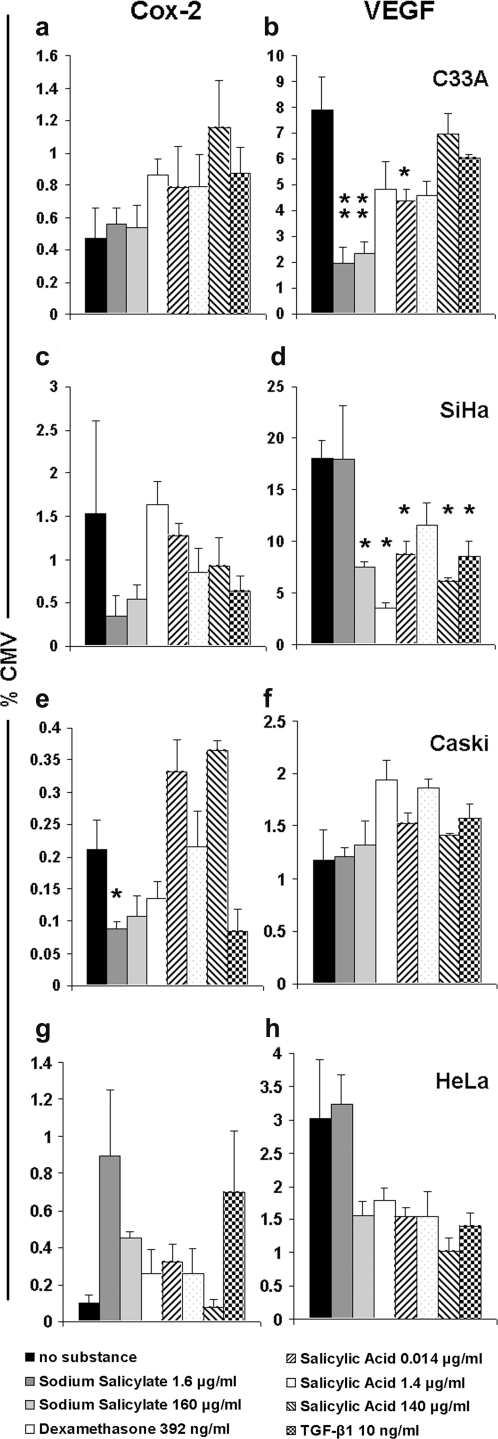
Regulation of Cox-2 and VEGF promoter activity with anti-inflammatory reagents. Monolayers were preincubated with reagents, and C33A (a, b), SiHa (c, d), Caski (e, f) and HeLa (g, h) cells were infected with 1 000 viral particles (vp)/cell of Ad5luc1 (with the CMV controlling luciferase), Adcox-2Mluc or AdVEGFluc, and luciferase expression was analyzed. Transgene expression level with Cox-2 and VEGF promoters are compared to the CMV promoter (%). Each point represents the mean of four experiments±standard error. *P<0.05, **P<0.01, ***P<0.0001 *versus* no substance.

The transcriptional activity of the Cox-2 and VEGF promoters was evaluated in cervical cancer cell lines with and without anti-inflammatory reagents sodium salicylate, dexamethasone, salicylic acid and TGF-β1 (Fig. 2a–h). Ad5luc1, which contains a very strong CMV promoter, was used for comparison, and relative luciferase activities are shown. Overall, the VEGF promoter induced a higher level of transgene expression than Cox-2 (Fig. 2b, d, f, h). Promoter expression was well in accord with previous data on Cox-2 and VEGF mRNA expression in these cell lines [19]. Although both promoters could be downregulated with anti-inflammatory substances, VEGF was more regularly affected (Fig. 2b, d).

### Oncolytic adenoviruses displayed efficient killing of cervical cancer cells *in vitro*

**Figure 3:**
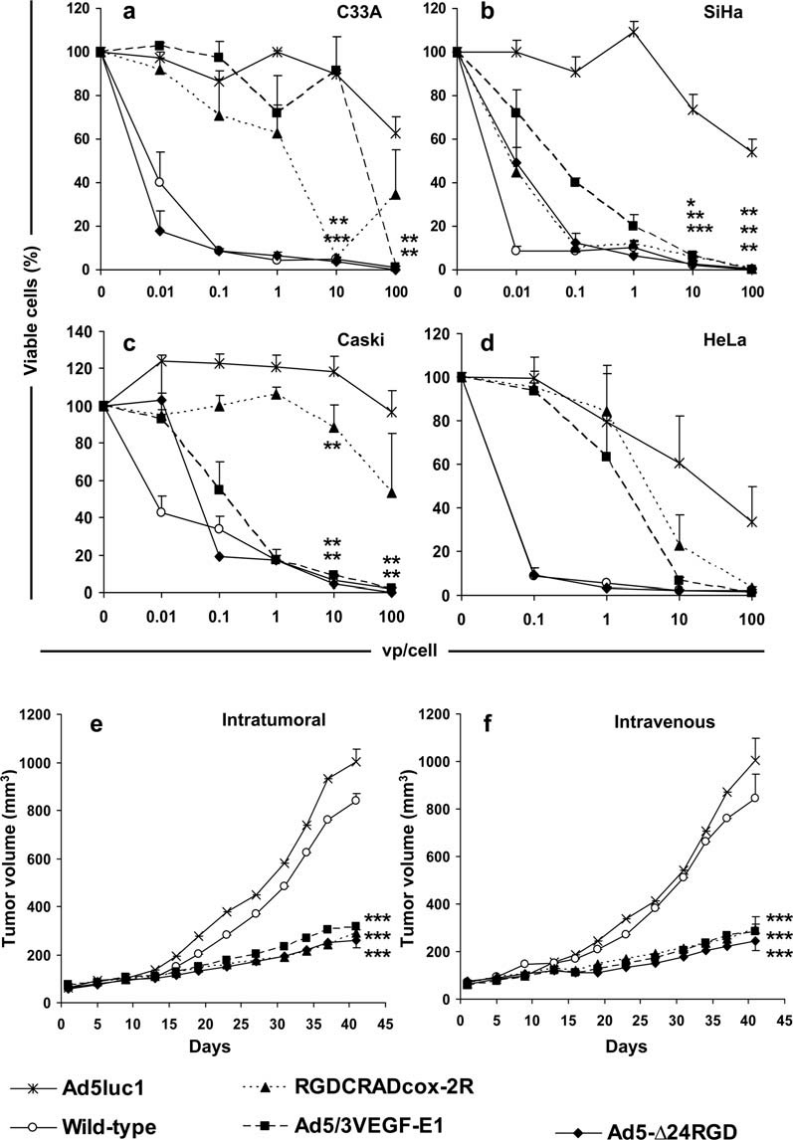
Oncolytic adenoviruses display efficient killing of cervical cancer cells *in vitro* and *in vivo*. (a–d) Monolayers were infected with RGDCRADcox-2R, Ad5/3VEGF-E1, Ad5-Δ24RGD, wild-type adenovirus and Ad5luc1 (*E1*-deleted control virus). Cell viability was measured with MTS assay. The OD_490_ values of uninfected cells were set as 100%. Data is expressed as mean±standard error of quadruplicate experiments. (e–f) C33A cells were injected subcutaneously into nude mice and advanced tumors were allowed to develop. The mice were treated either with (e) three intratumoral injections of 1×10^9^ viral particles (vp) of Ad5luc1, wild-type virus or oncolytic adenoviruses on three consecutive days, or with (f) a single intravenous injection of 1×10^11^ vp. *P<0.05, **P<0.01, ***P<0.0001 *versus* Ad5luc1. Bars indicate standard error.

Monolayers of cervical cancer cells were infected with oncolytic adenoviruses, wild-type virus and Ad5luc1, an *E1*-deleted control virus (Fig. 3a–d). In all cell lines, the quantitative cell killing assay showed cytolysis with oncolytic viruses and wild-type virus, while Ad5luc1 caused minimal cell killing. On most cell lines, oncolysis was significantly improved with replicating viruses in comparison to Ad5luc1. Further, oncolysis caused by RGDCRADcox-2R was significant also on C33A and Caski cells when dose of 10 vp/cell was used (Fig. 3a, c). On all cell lines, cell killing with Ad5-Δ24RGD was comparable to wild-type adenovirus, while the efficacy of RGDCRADcox-2R and Ad5/3VEGF-E1 was weaker.

### Oncolytic adenoviruses delivered therapeutic efficacy in murine cervical cancer models *in vivo*

Advanced subcutaneous C33A tumors were treated with three intratumoral injections of 1×10^9^ vp of Ad5luc1, wild-type virus, Ad5/3VEGF-E1, RGDCRADcox-2R or Ad5-Δ24RGD on three consecutive days, or with a single intravenous injection of 1×10^11^ vp of the same viruses. Treatment with oncolytic viruses gave significant therapeutic efficacy in both models (Fig. 3e, f: *P*<0.0001 for RGDCRADcox-2R, Ad5/3VEGF-E1 or Ad5-Δ24RGD *versus* Ad5luc1). Wild-type adenovirus did not display a significant effect on tumor growth (*P* = 0.1471 and 0.8297 *versus* Ad5luc1 for intratumoral and intravenous models, respectively).

### Anti-inflammatory reagents reduced oncolysis caused by Cox-2 and VEGF promoter driven oncolytic adenoviruses and wild-type adenovirus

**Figure 4:**
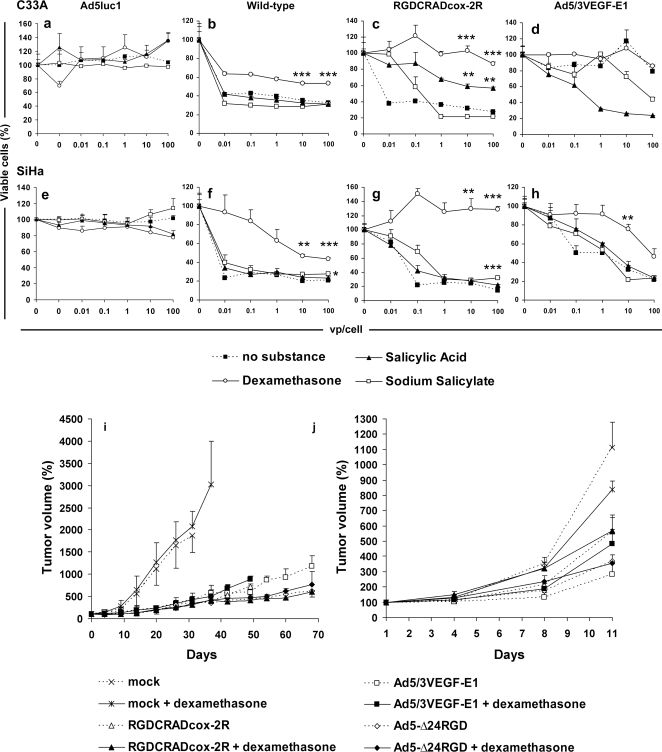
Dexamethasone and sodium salicylate reduces oncolysis caused by Cox-2 and VEGF promoter driven oncolytic adenoviruses and wild-type adenovirus *in vitro* but not *in vivo*. (a–h) Cell monolayers were preincubated with substances, and C33A (a–d) and SiHa (e–h) cells were infected with Ad5luc1, wild-type virus, Ad5/3VEGF-E1 and RGDCRADcox-2R. Cell killing efficacy of replicating viruses was reduced with dexamethasone (b, c, f–h) and sodium salicylate (c, f, g). To show the effect of the substances *per se* on cell survival, a second mock has been added in panels a and e. The y-axis crossing point indicates cell viability without virus or substances, while the “0” to the right of it indicates cell survival without virus but with substances. The *in vivo* effect of dexamethasone on therapeutic efficacy of oncolytic or wild-type adenoviruses. (i) Subcutaneous C33A cervical cancer tumors were allowed to establish and mice were treated with a single intravenous injection of 1×10^11^ viral particles (vp) of oncolytic viruses or no virus. In the RGDCRADcox-2R groups, 3×10^8^ vp were injected intratumorally on days 1, 3 and 5. In addition, the mice received intraperitoneal dexamethasone or PBS. (j) Human ovarian cancer (Hey) tumors were established in nude mice, and treated with intratumoral injections of 3×10^8^ vp of RGDCRADcox-2R, Ad5/3VEGF-E1, Ad5-Δ24RGD or no virus on days 1, 3 and 5. Mice received intraperitoneal injections of PBS or dexamethasone daily. Bars indicate standard error. Despite a suggestive trend at some time points, dexamethasone did not affect the growth of tumors significantly in overall analysis of either model. *P<0.05, **P<0.01, ***P<0.0001 *versus* no substance. Bars indicate standard error.

The effect of anti-inflammatory agents on oncolytic adenoviruses and wild-type virus was analyzed on C33A and SiHa cell monolayers. None of the analyzed reagents (dexamethasone, salicylic acid and sodium salicylate) caused significant cell killing on their own or in combination with replication deficient *E1*-deleted Ad5luc1 (Fig. 4a, e). The cell killing efficacy of replicating viruses was reduced with dexamethasone (Fig. 4b, c, f–h), salicylic acid (Fig. 4c) and sodium salicylate (Fig. 4f, g).

### The *in vivo* effect of dexamethasone on therapeutic efficacy of oncolytic or wild-type adenoviruses

Subcutaneous C33A cervical cancer tumors were allowed to develop and the mice were treated with a single intravenous injection of 1×10^11^ vp of Ad5/3VEGF-E1, Ad5-Δ24RGD or no virus. In the RGDCRADcox-2R groups, 3×10^8^ vp were injected intratumorally on days 1, 3 and 5. Then the mice were randomized to intraperitoneal dexamethasone or PBS treatment (Fig. 4i). Ad5-Δ24RGD was used as a model of an oncolytic virus without a tissue specific promoter. Wild type virus could not be used because it did not yield any efficacy in the model (Fig. 3). Despite some promising albeit minor trends, dexamethasone did not affect tumor growth significantly (*P* = from 0.5726 to 0.9909 *versus* virus only). However, all oncolytic adenoviruses continued to display anti-tumor efficacy as in the previous experiment (all *P*<0.0001 *versus* mock).

To see if we could tease out the replication attenuating effect of dexamethasone in a fast growing, highly aggressive subcutaneous model, we used Hey ovarian cancer cells (Fig. 4j). Previous work suggested that the viruses used here would replicate in Hey cells [36], [37]. Xenografts were treated with intratumoral injections of 3×10^8^ vp of viruses or no virus on days 1, 3 and 5. Mice received intraperitoneal injections of PBS or dexamethasone daily, and tumor growth was followed. Again, although there was a suggestion of attenuation of virus replication (*i.e.* larger tumors), dexamethasone had no significant effect on therapeutic efficacy of analyzed viruses (*P* = from 0.8897 to 0.9441). The antitumor efficacy of oncolytic adenoviruses continued to be significant compared to mock-treatment (all *P*<0.0001).

### The effect of dexamethasone on replication of Cox-2 and VEGF promoter driven oncolytic adenoviruses and wild-type adenovirus on cervical cancer cells *in vitro*

**Figure 5:**
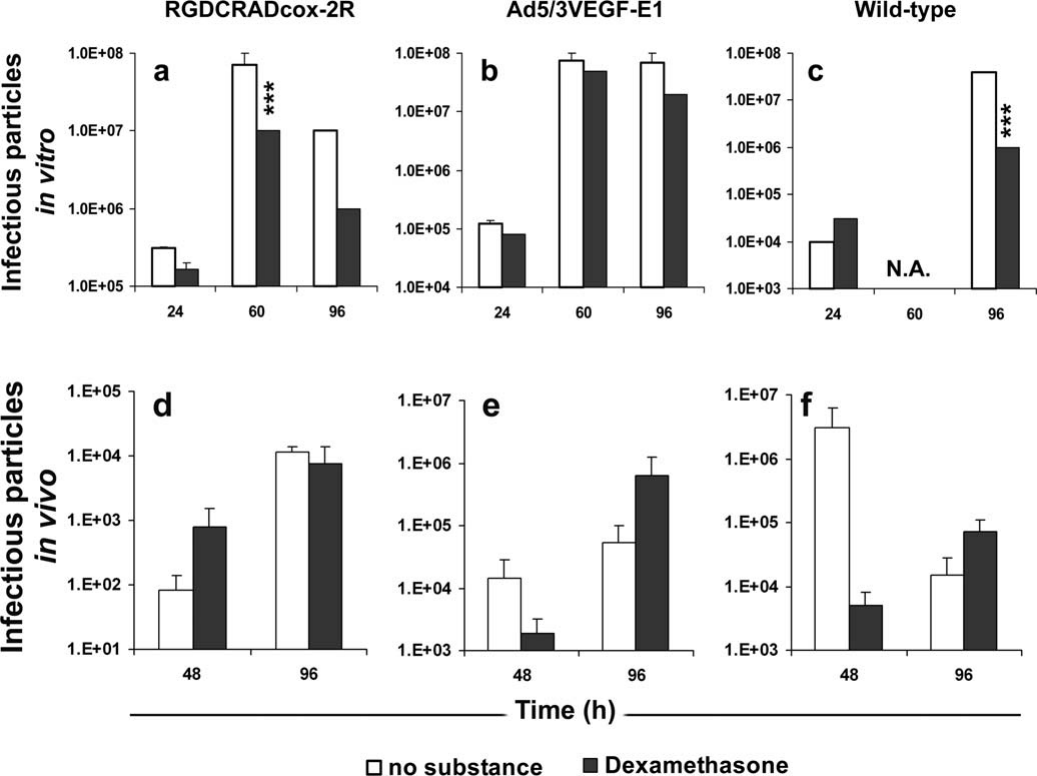
Dexamethasone reduces replication of oncolytic adenoviruses Ad5/3VEGF-E1, RGDCRADcox-2R and wild-type adenovirus *in vitro* but not *in vivo*. (a–c) SiHa cervical cancer monolayers were infected with viruses alone (10 viral particle (vp)/cell) or in combination with dexamethasone, and virus production was analyzed by plaque assay. Despite a clear trend at early time points, there was no statistically significant effect of dexamethasone on *in vivo* replication due to larger variation *in vivo* (d–f). Human ovarian cancer tumors were established in nude mice and treated with a single intratumoral injection of 3×10^8^ vp. Mice received intraperitoneal injections of dexamethasone or PBS daily. Four tumors/group were harvested on days 2 and 4, and the amount of infectious particles was analyzed by TCID_50_. ***P<0.0001 *versus* virus alone. N.A. = not analyzed. Bars indicate standard error.

We analyzed the *in vitro* production of virions by RGDCRADcox-2R, Ad5/3VEGF-E1 and wild-type adenovirus in SiHa cells with and without dexamethasone treatment (Fig. 5a–c). Overall, dexamethasone reduced the replication of analyzed viruses. Replication of RGDCRADcox-2R was reduced 2-, 7-, and 10-fold at 24, 60 and 96 h, respectively (Fig. 5a: *P*<0.0001 at 60 h). A similar but weaker effect was seen with Ad5/3VEGF-E1 (Fig. 5b: 1.5 to 3.5-fold, *P* = 0.0640 at 96 h). Replication of a wild-type virus was significantly reduced at 96 h (Fig. 5c: 40-fold, *P*<0.0001).

### The effect of dexamethasone on replication of Cox-2 and VEGF promoter driven oncolytic adenoviruses and wild-type adenovirus on cancer cells *in vivo*

Regulation of replication by dexamethasone *in vivo* was analyzed with the subcutaneous human ovarian Hey adenocarcinoma tumors treated with intratumoral injections on day 0. Half of the mice received intraperitoneal injections of dexamethasone daily. Tumors were analyzed on days 2 and 4. Although dexamethasone reduced the replication of Ad5/3VEGF-E1 and wild-type virus at early time point, the difference was not statistically significant (Fig. 5d–f, all *P* = 0.1283–0.6144).

## Discussion

Because the primary adenovirus receptor may be frequently absent or expressed aberrantly in advanced tumors [34], we first analyzed *in vitro* transduction efficacy of fiber modified, infectivity enhanced adenovirus vectors expressing the luciferase transgene (Fig. 1). RGD-4C modification did not seem to be very effective in increasing gene transfer, but the Ad3 receptor retargeted virus was quite effective in three out of four cell lines (Fig.1a–d). Further, we evaluated the effect of the anti-inflammatory reagents on transduction efficacy, and found some increase in transgene expression after treatment with dexamethasone on two out of four cervical cancer cell lines (Fig.1 f,g) As a major determinant of the efficacy of oncolytic adenoviruses is infectivity [34], we utilized genetic fiber modifications for improving cell killing efficacy. We evaluated the *in vitro* cytolytic potency of these oncolytic adenoviruses in cervical cancer cell lines, and found correlation between oncolytic potency, gene delivery and promoter activity, *i.e.* the stronger the promoter and higher the infectivity, the stronger was the oncolytic potency (Fig. 3a–d).

More importantly, all the analyzed oncolytic adenoviruses had statistically significant therapeutic efficacy in both local and systemic treatment schemas of murine cervical cancer xenografts (Fig. 3e, f). Some variation in efficacy was seen between *in vitro* and *in vivo* results with some of the viruses. This may due to recent discoveries suggesting that while gene delivery *in vitro* depends mostly on primary adenovirus receptors, bioavailability issues seem to dominate with regard to *in vivo* efficacy. For example, it is increasingly accepted that while binding to CAR is an important determinant of transduction *in vitro*, other regions of the fiber may be even more relevant *in vivo* [38]. In parallel to previous findings with other cell lines [37], [39], wild type adenovirus was not effective on C33A cells *in vivo*, despite activity *in vitro*. Although we assume this relates to differences between *in vitro* and *in vivo* environments (eg. stroma, vasculature, receptor expression), further work is needed to clarify the issue.

When Cox-2 and VEGF promoter driven transgene expression was evaluated, both promoters were found active in cervical cancer cell lines. Overall, VEGF promoter activity was higher than Cox-2 in all cell lines, and comparable to previous data [19]. Importantly, earlier studies have reported that normal liver cells do not express VEGF [40]. Also Cox-2 levels seen in cervical cancer cells were higher than what has been reported for the liver previously [16]. Significant reduction of VEGF promoter mediated luciferase expression was seen with sodium salicylate, dexamethasone and salicylic acid (Fig. 2b, d). Sodium salicylate also reduced Cox-2 promoter controlled transgene expression (Fig. 2e).

When cell killing experiments were performed in the presence of anti-inflammatory agents, dexamethasone and sodium salicylate were effective in reducing oncolysis (Fig. 4). Interestingly, the effect was not restricted to oncolytic adenoviruses, but also wild type virus displayed weaker cytolysis when dexamethasone was present. Thus, the effect might not be completely related to the promoter controlling the replication, but a more general phenomenon in viral replication might be also involved. One cause of reduced replication might be down-regulation of the relevant receptors required for infection. Previously, we and others have analyzed the modulation of adenovirus primary receptor expression on the cell surface by various substances including a number of chemotherapeutics and anti-inflammatory reagents. We found no effect on receptor level, as assessed by flow cytometry analysis, after dexamethasone treatment, while others detected a slight reduction in the level of both primary receptor and α_v_β integrins [41], [42].

The effect of dexamethasone on the serotype 3 receptor had not been studied, nor had the cell lines used here been studied before with regard to the other relevant adenovirus receptors. We therefore analyzed the effect of the substances on gene delivery and found that in some cases luciferase expression was increased (Fig. 1e–h). Thus, the reduced replication seen here was probably not due to receptor downregulation.

Another mechanistic possibility might involve induction of Cox-2 by virus replication *per se*. With regard to herpes, cytomegalovirus, and other DNA viruses, it has been demonstrated that virus infection induces Cox-2 [43], [44]. Further, the finding that inhibition of Cox-2 reduces replication of these viruses suggests that Cox-2 induction is beneficial for virus propagation. These viruses may utilize the anabolic effects of Cox-2 for optimization of their replication efficacy. Preliminary data suggests that the same may also be true for adenovirus, which might help explain why the oncolytic effect of wild-type adenovirus was attenuated by dexamethasone [45].

Although oncolysis is likely to correlate with replication of the virus, we investigated this separately. As expected, virus replication was reduced with dexamethasone treatment *in vitro* (Fig. 5a–c). This seems to support the theoretical assumption that oncolysis is tightly linked with virus replication. As human adenoviruses do not replicate productively in murine normal tissues [46], human xenografts in mice were utilized for replication attenuation *in vivo* studies. In these models, if replication and/or cell killing efficacy is reduced *in vivo* with dexamethasone, tumors in mice treated with virus and dexamethasone would be larger than virus only treated. In both models studied, dexamethasone did not significantly reduce the antitumor efficacy of the analyzed oncolytic adenoviruses, despite a trend in that direction (Fig. 4i–j, all *P*≥0.1654). Finally, we analyzed the amount of infectious particles in subcutaneous tumors with and without dexamethasone treatment (Fig. 5d–f). Despite a trend prominent at early time points, no significant differences were seen, which may be due to variation typical of *in vivo* experiments.

The most likely reason for the discrepancy between the observed *in vitro* and *in vivo* effect of dexamethasone on the oncolytic potential of the viruses may relate to the higher complexity of *in vivo* models. These complexities were well demonstrated in a recent study where an increase in VEGF levels in Cox-2 positive and Cox-2 negative pancreatic cancer cells was seen after treatment with high concentrations of Cox-2 inhibitors, suggesting that the relationship between Cox-2 protein inhibition and VEGF or Cox-2 promoter expression may not always be tightly linked [47]. Contrary to expectations, both Cox-2 positive and negative *in vitro* models displayed increased levels of VEGF following Cox-2 inhibition. However, in the Cox-2 positive tumor *in vivo* model, non-malignant cells expressed a markedly decreased level of murine VEGF leading to reduced total VEGF and tumor angiogenesis and growth, while Cox-2 negative tumors displayed increased tumor growth. These results also suggest that the tumor stroma may have a major effect on the expression of Cox-2 and related factors.

Another aspect relates to the non-linear relationship between E1A levels and efficacy of virus replication. Classic studies suggest that highly variable E1A levels allow effective replication without direct correlation between E1A expression and virion production [48]. Thus, is it quite possible, that even though E1A expression was affected due to dexamethasone inhibiting the promoters, the effect was not dramatic enough to be seen as a difference in tumor growth curves. Dexamethasone regulates multiple components of both innate and adaptive immunity. The nude mice used in the study lack functional T cells, but possess normal B cells, NK cells, macrophages etc. Innate immunity is important for clearance of adenovirus and therefore it is possible that the effects of the drug on virus and on the remaining immune system neutralized each other, thus showing no significant differences.

Adenoviruses can cause severe toxicity in immunocompromized individuals. Although clinical trials in cancer patients have heretofore reported extremely good safety data [6], [7], preclinical work suggests that there is the potential for toxicity [49]. Further, most oncolytic adenovirus trials completed have utilized early generation viruses, which are rather attenuated in their replicative potential. Thus, increasingly effective oncolytic adenoviruses could result in more toxicity and therefore it would be useful if replication could be abrogated if necessary. The data presented here suggests that anti-inflammatory reagents dexamethasone and sodium salicylate can reduce the activity of Cox-2 and VEGF promoters. Further, this resulted in reduced replication and oncolytic potential of the respective replicative viruses *in vitro*. The effective doses were well within what would be predicted safe in humans based on published trials [6], [7], [50], [51].

Dexamethasone is routinely administered to cancer patients as an anti-emetic or because of its anti-inflammatory, anabolic and psycho-stimulating effects [52]. Dexamethasone use is particularly prevalent in end-stage cancer patients, who could be candidate for experimental approaches such as oncolytic viruses. This suggests that it might be useful to address dexamethasone use in trial protocols featuring agents that utilize the Cox-2 or VEGF promoters. Moreover, if it is confirmed that dexamethasone and/or other anti-inflammatories reduce adenovirus replication and efficacy *per se*, this should be taken into account in all oncolytic adenovirus trials. On the other hand, this phenomenon certainly might be useful for intervention in case of side effects in trials. Further, abrogation of replication could be useful in the rare cases of dangerous adenovirus infections in immunosuppressed, transplant and pediatric patients. Finally, the effect on anti-inflammatories on Cox-2 promoter or protein levels, and their association with virus replication, could help shed light on adenovirus biology, and the interactions between human cells and adenoviruses.

## Methods

### Cell lines and agents

Caski, C33A, SiHa and HeLa cervical cancer and A549 lung adenocarcinoma cell lines were obtained from ATCC (Manassas, VA). 293 cells were purchased from Microbix (Toronto, Canada). Ovarian adenocarcinoma Hey cells was obtained from Dr. Wolf (M.D. Anderson Cancer Center, Houston, TX). Dexamethasone, Sodium Salicylate, Salicylic Acid and TGF-β1 were purchased from Sigma (St. Louis, MO). The concentrations used correspond to achievable, bioactive and well tolerated concentrations in human serum following treatment with the agents, as indicated by the results of a comprehensive literature search.

### Adenoviruses

The viruses utilized in the experiments are listed in Table 1. *E1*-deleted viruses were propagated on 293 cells, while replicating viruses were propagated on A549 cells. Viruses were purified on cesium chloride gradients. The vp concentration was determined at 260 nm, and plaque assay was performed to determine infectious particles [37].

**Table 1:** Viruses used in the experiments.

Virus	E1A	Reporter	Fiber	Main receptor*	Ratio†	Ref
**Ad5luc1**	deleted	luciferase	wild type	CAR	5.4	[53]
**Ad5/3luc1**	deleted	luciferase	serotype 3 knob	CD46 and unknown	5.0	[54]
**Ad5lucRGD**	deleted	luciferase	RGD motif in HI-loop	α_v_β integrins and CAR	53	[55]
**Adcox2Mluc**	deleted	luciferase	wild type	CAR	60	[16]
**AdVEGFluc**	deleted	luciferase	wild type	CAR	67	[18]
**Ad5-Δ24RGD**	24 bp deletion	-	RGD motif in HI-loop	α_v_β integrins and CAR	39	[33]
**RGDCRADcox-2R**	heterologous Cox-2 promoter controlling E1A expression	-	RGD motif in HI-loop	α_v_β integrins and CAR	8.5	[56]
**Ad5/3VEGF-E1**	heterologous VEGF promoter controlling E1A expression	-	serotype 3 knob	CD46 and unknown	20	[24]
**Ad300wt = wild type human Ad5**	wild type	-	wild type	CAR	10	ATCC

* CAR = coxsackie-adenovirus receptor.

† ratio of viral particles (vp) to plaque forming units (pfu), a quality control measure and indicator of viral packaging efficacy.

### Adenovirus-mediated gene transfer assays

Cells were infected for 30 min, washed once, and complete medium was added. After 24 h incubation, luciferase assay was performed (Luciferase Assay System, Promega, Madison, WI). The protein concentration was determined using a Bio-Rad DC protein assay kit (Bio-Rad, Hercules, CA). Background luciferase activities were subtracted from the readings. In order to analyze the effect of anti-inflammatory reagents on transduction efficacy, Dexamethasone (392 ng/ml), Sodium Salicylate (160 µg/ml) and Salicylic Acid (1.4 µg/ml) were added 18 h prior the infection, and the infection and incubation were performed in the presence of the substances. These doses did not cause toxicity to cells.

### The effect of anti-inflammatory reagents on promoter activity

Reagents were added 18 h prior the infection, and the infection with Ad5luc1, Adcox-2Mluc or AdVEGFluc, and incubation were performed in the presence of the substances. Luciferase expression was analyzed as above. Transgene expression levels with Cox-2 and VEGF promoters are compared to CMV promoter, and relative luciferase activities are shown. The results without reagents were compared to the other groups. All comparisons were conducted with a Student's t-test with Satterthwaite's approximation for unequal variances if indicated (SAS v.9.1, SAS Institute, Cary, NC). For all analyses a two-sided *p* value of <0.05 was deemed statistically significant.

### Cell killing assays

Cells in quadruplicate were infected with Ad5luc1, wild-type, Ad5/3VEGF-E1, RGDCRADcox-2R and Ad5-Δ24RGD. Thereafter, cells were incubated with complete growth medium. Cell viability was measured using the MTS assay (Promega) when any virus at 10 vp/cell displayed complete cell killing. The results with Ad5luc1 and wild-type were compared to the other groups using two-tailed *t*-test as above. The effect of anti-inflammatory agents on cell killing efficacy was analyzed on C33A and SiHa cells.

### Regulation of replication by dexamethasone *in vitro*

Cells were infected with viruses alone or in combination with dexamethasone, and replication was analyzed after three freeze-thaw cycles by plaque assay. The effects of dexamethasone was analyzed using bootstrap multiple comparisons of means (PROC MULTTEST SAS v9.1). The levels of viral replication were log transformed for normality. A multiplicity adjusted bootstrap *p* value of <0.05 was deemed statistically significant.

### *In vivo* cancer models

All animal protocols were reviewed and approved by the Experimental Animal Committee of the University of Helsinki and the Provincial Government of Southern Finland. In efficacy experiment, mice were obtained from Charles River Laboratories (Wilmington, MA) and subcutaneous tumors were established by injecting 10^7^ C33A cells into female nu/nu mice. 1×10^9^ vp of Ad5luc1, wild-type, Ad5/3VEGF-E1, RGDCRADcox-2R, Ad5-Δ24RGD, or no virus, were injected intratumorally on days 1, 2 and 3 (*n* = 5 mice, 10 tumors/group). Another group of mice received the virus intravenously as a single injection of 1×10^11^ vp on day 1 (*n* = 4 mice, 8 tumors). Tumor size was measured.

In the *in vivo* regulation assay, mice were obtained from Taconic (Ejby, Denmark), subcutaneous C33A cell tumors were established as above, and treated with a single intravenous injection of 1×10^11^ vp on day 1. To study the effect of a different route of administration, 3×10^8^ vp were injected intratumorally on three consecutive days in the RGDCRADcox-2R groups. Mice received intraperitoneal injections of PBS or dexamethasone (50 µg) daily (*n* = 6 mice, 12 tumors/group). Tumor size was followed. 5/12 mice receiving intravenous Ad5/3VEGF-E1 treatment died within 12 h. Dexamethasone treatment did not affect toxicity. Livers were harvested and fixed in buffered formalin. Histopathology did not reveal any liver toxicity (data not shown).

Subcutaneous human ovarian cancer (Hey) tumors were established in female NMRI CD-1 nude (*n* = 5 mice, 10 tumors/group), and treated with intratumoral injections of 3×10^8^ vp on days 1, 3 and 5. Mice were treated with dexamethasone as above.

Regulation of replication by dexamethasone *in vivo* was analyzed with the Hey cell tumors treated with a single intratumoral injection. Half of the mice received dexamethasone. 4 tumors/group were harvested on days 2 and 4, weighted, homogenized, and the virions were released by three freeze-thaw cycles. The amount of infectious particles was analyzed by TCID_50_. The analysis of the tumor size data was performed using a repeated measures growth model with PROC MIXED (SAS v.9.1), which treated the within mouse effect of time as a continuous variable and the treatment group as a fixed effect. The tumor size data was log transformed. The effects of treatment group, time and the interaction of treatment group and time were evaluated by *F* tests. Baseline tumor size was included as a covariate in all models and flank as a covariate. The *a priori* planned comparisons of differences in predicted treatment means were computed by *t*-statistics at study's end and averaged over all time points. Tukey-Kramer adjustment was utilized to allow for multiple comparisons.
